# Pediatric Chronic Pain, Resilience and Psychiatric Comorbidity in Canada: A Retrospective, Comparative Analysis

**DOI:** 10.3389/frhs.2022.852322

**Published:** 2022-05-03

**Authors:** Megan A. Young, Polina Anang, Anna Gavalova

**Affiliations:** ^1^Department of Psychiatry, Max Rady College of Medicine, University of Manitoba, Winnipeg, MB, Canada; ^2^Department of Neuroscience, Faculty of Science, University of Winnipeg, Winnipeg, MB, Canada

**Keywords:** pediatric, chronic pain, resilience, psychiatric disorder, comorbidity, prosocial behavior, somatic symptom and related disorders, somatic symptom disorder

## Abstract

**Objective:**

Chronic pain compromises child and adolescent well-being and development. This study aimed to identify risk factors for chronic pain and exploration of how young people negotiate such risks and express resilience. We hypothesized children and youth with chronic pain would report greater prevalence of mental health disorders than the general population; and those demonstrating greater resilience would demonstrate less psychiatric comorbidity.

**Method:**

A retrospective chart review was conducted for all patients (ages 7–17) attending the sole pediatric chronic pain clinic in Manitoba, from 2015 to 2019 (*N* = 116). Patients' demographic information and psychiatric illness burden were compared to provincial epidemiological data using Chi-Square tests. Pain sites, family history, psychiatric illness, psychosocial functioning, treatment history and treatment recommendations were explored.

**Results:**

The sample was predominantly female (74%; *N* = *114*). Sixty-eight percent of patients reported a family history of chronic pain. Thirty-seven percent of the patients (vs. 14.0% anticipated; *N* = 326 260) reported comorbid psychiatric disorder, X^2^ (1, *N* = 114) = 53.00, *p* < 0.001. Thirty-two percent reported diagnosis of mood and/or anxiety disorder (vs. 7.3%), X^2^ (1, *N* = 114) = 99.34, *p* < 0.001. Children and youth demonstrating resilience through engagement in more prosocial behaviors reported fewer psychiatric symptoms (rs = −0.292*, N* = 114, *p* = 0.002, Spearman's correlation).

**Conclusions:**

Female sex, family history, and lower socioeconomic status were associated with chronic pain. Psychiatric conditions were more prevalent in chronic pain patients than in the general population. Approaching chronic pain from a mind-body perspective, while building on patients' strengths, is central to informing treatment.

## Introduction

Chronic and recurrent pain is detrimental to the well-being and development of children and youth (1). It is estimated that 5–15% of children and adolescents experience severe chronic pain and/or pain-related disability (2). Worldwide estimates of 10–20% of the general pediatric population have a diagnosis of a psychiatric disorder, with anxiety and depression being most common (3). There is evidence of a relationship between these conditions in psychiatric patients. Within a large European health survey, 70% of pediatric patients with previous diagnosis of psychiatric disorders reported symptoms of chronic pain (4). Conversely, there is limited evidence in the literature regarding chronic pain patients' experience of psychiatric comorbidity.

Chronic pain has been acknowledged as a disease in its own right, with emphasis on psychosocial implications. The World Health Organization defines primary chronic pain within the 11th Revision of the International Classification of Diseases (ICD-11) as “persistent or recurrent pain that has persisted for more than 3 months and is associated with significant emotional distress and/or functional disability, and the pain is not better accounted for by another condition” (5). They also define chronic secondary pain where pain may be conceived as a symptom secondary to an underlying disease but persists even after the condition has been treated (5). Within the pediatric chronic pain literature, chronic pain is usually defined temporally, as persistent or recurrent pain of greater than or equal to 3 to 6 months duration (1).

Individuals referred to a tertiary chronic pain clinic are generally experiencing persistent and medically unexplained pain, or low levels of underlying pathology that does not explain the presence and extent of pain, or both (6). They are more likely to have severe pain and pain-related disability that has a wider, bidirectional relationship with social and physical functioning, family life, and parenting (7). In essence, children and youth who attend a tertiary chronic pain clinic may meet the criteria for both an ICD-11 chronic pain diagnosis as well as a Somatic Symptom Disorder with predominant pain as defined within the Diagnostic and Statistical Manual of Mental Disorders-Fifth Edition (DSM-5). The DSM-5 criteria for somatic symptom disorder with predominant pain applies to those with chronic pain when it has persisted for longer than 6 months and there is disruption of daily life or distress. In addition, one or more criterium is required to establish the diagnosis: high levels of anxiety about symptoms, excessive energy devoted to health concerns, disproportionate and persistent thoughts about the seriousness of one's symptoms (8). In a retrospective chart review of pediatric patients hospitalized with somatic symptom and related disorders (SSRD) diagnosis, 97% reported pain as part of the SSRD (9).

The plausible etiology and risk factors for the development of severe chronic pain are complex. Children of parents with chronic pain may have an elevated risk of developing pain-related disability as a consequence of several composite interactions inclusive of genetic predisposition for heightened pain sensitivity, social learning and parental responses to pain, such as protectiveness and parental catastrophizing (10). Social factors, including exposure to childhood trauma and socioeconomic deprivation, may also increase the severity of chronic pain and pain-related disability (1, 11). There is also evidence of sex and gender differences in prevalence of chronic pain with co-existing psychiatric illnesses reportedly higher in adult females and increasing with age (12). The shared cognitive and behavioral factors, as well as neurobiological development and maintenance of these conditions has been extensively studied and reviewed (13).

Focus on the negative impact of chronic pain prevents us from paying sufficient attention to strengths and resilience. Resilience is conceptualized as responding with mastery when faced with adversity. It has been defined as evidence of inherent strength within the individual enhanced through psychosocial experiences (14). There is limited research of inherent strengths in youth affected by chronic pain. Measuring youth resilience is no easy task; notwithstanding, regular school attendance, academic achievement, participation in extracurricular activities, and engagement in prosocial behavior may be protective regarding the development of comorbid psychiatric conditions and pain-related disability and provide tangible evidence of resilience (15).

The purpose of the present study was to explore potential risk factors for development of moderate to severe chronic pain, psychosocial attributes, comorbid psychiatric symptoms and disorders, and resilience of children and adolescents who are experiencing chronic pain resulting in referral to tertiary care.

Specifically, the researchers hypothesized:

1) Children and adolescents with chronic pain will have greater psychiatric illness burden than the general population from which they were derived.
2) Those who demonstrate greater functional status, indicative of resilience, will have fewer associated psychiatric comorbidities.

## Methods

### Study Design

A retrospective chart review was conducted for all pediatric patients attending a Canadian pediatric chronic pain clinic located at a university medical center from October 2015 to September 2019. The clinical data was derived from the only tertiary pediatric chronic pain clinic located within the province of Manitoba, Canada, allowing for total population sampling of attendees over a four-year period. Aggregated data related to the population from which the sample was derived was also accessed for comparative analysis of demographic information and psychiatric illness using correlative analyses and Chi-squared tests.

### Study Sample

Inclusion criteria was defined as any child or youth under the age of 19 who attended the pediatric chronic pain clinic for assessment in Manitoba that met the diagnostic criteria of chronic pain as outlined in the ICD-11 definition of chronic pain between October 2015 and September 2019. The only two defined exclusion criteria were being older than 19 years of age and/or if the pediatric patient did not meet the criteria for an ICD-11 definition of chronic pain. No other exclusion criteria were defined.

Children and youth were referred to the clinic by Manitoba- based physicians from primary care, emergency, as well as other specialist clinics. One-hundred and sixteen charts, including all youth under the age of 19 with first visit to the chronic pain clinic, were reviewed (ages 7–17; *n* = 116). Two subjects were excluded from further analysis as they did not meet the ICD-11 diagnostic criteria for either primary or secondary chronic pain (*n* = 114).

### Variables and Outcomes of Interest

**Figure 1:**
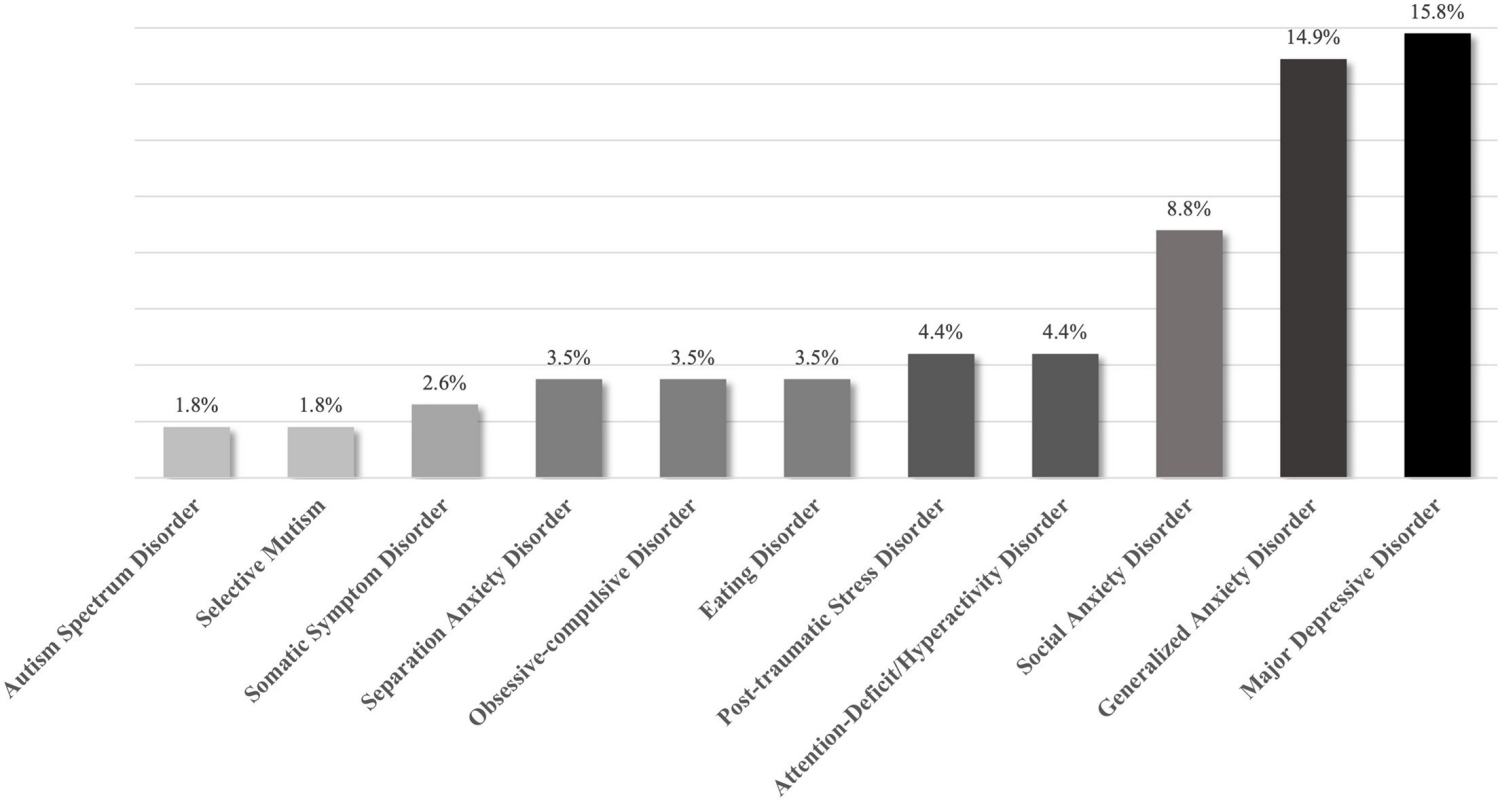
Prevalence of previously diagnosed mental health disorder reported by pediatric chronic pain patients (*n* = 114).

**Figure 2:**
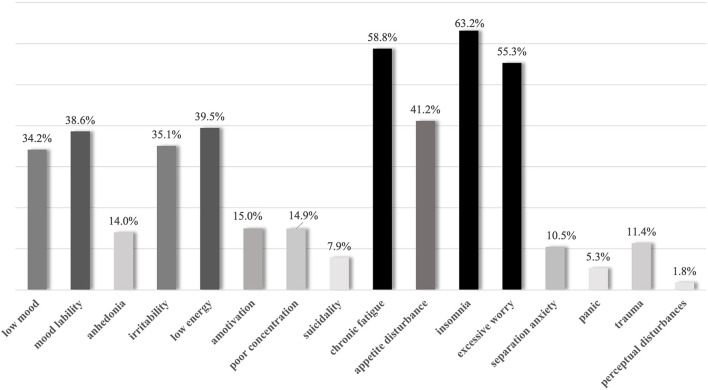
Prevalence of reported psychiatric symptoms in children youth diagnosed with chronic pain (*n* = 114).

Variables measured included age, sex, pain location(s) and type, family history of first and second degree relatives with chronic pain and/or psychiatric disorder, comorbid psychiatric symptoms and diagnoses (see Figures 1, 2), resiliency markers, treatment history and treatment recommendations. Patient or caregiver report of the patient experiencing psychiatric symptoms of anxiety, depression, and post-traumatic stress disorder (PTSD), as well as those meeting the DSM-5 based criteria for formal diagnoses of any mental disorder were also recorded. The researchers sought written chart evidence of continued participation in regular, healthy, and goal directed activities as a measure of resilience. Resilience was quantified through continued prosocial activities, including attending school regularly (absent < 10% of school days), as well as engaging in extracurricular activities, social interaction outside of the home, or working or volunteering (for those ages 14 and older) one time or more per week.

### Data Extraction

All information was de-identified when collected from the clinical charts in electronic form within a password-encrypted spreadsheet by two members of the research team. Demographic information related to age, sex, and first three digits of postal code were recorded verbatim. Pain location(s) and type, family history of first and second degree relatives with chronic pain and/or psychiatric disorder, comorbid psychiatric symptoms and diagnoses (see Figures 1, 2), resiliency markers, treatment history and treatment recommendations were recorded in a dichotomous format (i.e., yes/no).

The clinical data was derived from the only tertiary pediatric chronic pain clinic located within the province of Manitoba, Canada, providing capacity for total population sampling of attendees over a 4-year period. This information was then compared using Chi-Squared tests to the aggregated data contained within The Manitoba Center for Health Policy (MCHP) issued report in 2016 of 4-year period prevalence of mental and developmental disorders and suicidal behavior in the pediatric population of Manitoba (ages 0–19; *N* = 326 260). The MCHP Repository has been extensively validated for the use of epidemiological and social services research (16). The MCHP data is culminated from the Winnipeg Regional Health Authority, Manitoba Education and Training, Healthy Child Manitoba, Manitoba Families, Manitoba Justice, Manitoba Adolescent Treatment Center, and the Canadian Vital Statistics Agency (17). The detailed demographic information of the population and their inclusion criteria for mental health disorders are available online within the open-access report (17).

### Statistical Analysis

Chi-square tests were performed to determine whether the prevalence of *psychiatric diagnoses* for children and youth diagnosed with chronic pain were equal to those reported in the general population from which the sample was derived. The frequency and type of *psychiatric symptoms* reported by the clinical sample of children meeting the diagnostic criteria for chronic pain was also compared to the general pediatric population using a Chi-Squared test. Within the clinical sample, the other primary outcome of an association between demonstration of resilience (inferred through greater functional status) and less psychiatric symptom burden and/or confirmed psychiatric diagnosis were explored. Correlative analysis was also used to evaluate anticipated associations between psychiatric symptom burden and/or confirmed psychiatric diagnoses and age; associations between family history of mental illness, number of psychiatric symptoms endorsed and number of pain sites.

### Ethical Considerations

Ethical approval was obtained from the university and hospital research committee prior to study commencement. Consent was not obtained as all data was obtained retrospectively and was anonymized at time of collection (or in the case of the epidemiological data, as part of another larger study) and presented solely in aggregate form.

## Results

### Demographic Factors and Comparisons

The sample (*n* = 114) was predominantly female (74%) with an age range of 7 to 17 years old (M = 13.72; SD 2.4). A Pearson correlation was used to evaluate the association between psychiatric comorbidities and age; older children reported greater psychiatric symptom burden *r* (108) = 0.28, *p* = 0.003. There were no statistically significant correlations between sex and psychiatric or pain symptom burden, or age and indicators of resilience. Fifty-five percent of clinic attendees resided in a home with a median income below the poverty line, when the regional Market Basket Measure threshold was applied (M = $37107.91 CAD; SD = $6971.33 CAD) (18). A Spearman's correlation was used to evaluate the association between mental health diagnoses and socioeconomic status; lower household median income was associated with prior diagnosis of a psychiatric disorder (rs = −0.217, *n* = 114, *p* = 0.021). Sixty-eight percent of children and youth had reported a family history of chronic pain and 44% reported a family history of formal diagnosis of mental illness. There was no statistically significant association between family history of mental illness, number of psychiatric symptoms endorsed or number of pain sites.

Chi-square tests were performed to determine whether the prevalence of psychiatric diagnoses for children and youth diagnosed with chronic pain was equal to those reported in the general population from which the sample was derived. Thirty-seven percent of the clinical sample reported a previous diagnosis of a mental health disorder whereas 14% of all children and adolescents in the general population were given a diagnosis of any mental disorder (Chartier, 2016, X^2^ (1, *N* = 114) = 53.00, *p* < 0.001). Thirty-two percent reported a previous diagnosis of a mood and/or anxiety disorder (vs. 7.3%), X^2^ (1, *N* = 114) = 99.34, *p* < 0.001.

### Resilience and Symptom Severity

Less than half of the children were engaging in regular, prosocial behaviors indicative of resilience: 37.7% were attending school regularly, 44% were engaging in regular extracurricular activities, 40% of children were engaging in regular social activities, and 6% were working or volunteering. A Spearman's correlation was used to evaluate the association between resilience and psychiatric symptom burden. Children and youth engaging in prosocial behaviors indicative of higher levels of resilience reported fewer psychiatric symptoms (rs = −0.292, *n* = 114, *p* = 0.002).

### Pain and Psychiatric Symptom Burden

A Spearman's correlation was also used to evaluate the association between pain symptom burden and psychiatric symptom burden. Children and youth who reported a greater number of pain sites endorsed increased psychiatric symptoms (rs = 0.247, *n* = 114, *p* = 0.010, Spearman's correlation). Most common pain sites reported included musculoskeletal pain (78%), headache (69%); and abdominal pain (57%).

### Treatment History and Treatment Recommendations

Within the clinical sample, many children and youth had previous contact with psychiatric or psychological services (46%). Seventy-four percentage were encouraged to engage in previously accessed psychiatric or psychological treatment at time of initial assessment or were referred for psychiatric services by the chronic pain specialist. The majority of children were encouraged to engage in educational and prosocial activities, specifically, nearly all were encouraged to:

return to or continue to attend school (98%);
engage in prosocial activities, work, or volunteering (96%)

The vast majority of children and youth were provided some psychoeducation at time of assessment (97%) related to what chronic pain is and how it can be managed. Seventy-five percent of attendees were referred on for further assessment by a pediatric consult-liaison psychiatrist or psychological support within the clinical health psychology program with options for group and individual Cognitive Behavioral Therapy. Seventy percent were referred for physiotherapy or occupational therapy. Twenty-one percent of attendees were prescribed pharmacotherapy to target pain symptoms and pain-related psychological distress.

## Discussion

Chronic pain in children and youth is common (1). This study focused specifically on those who were referred to a tertiary care chronic pain clinic for diagnostic assessment and management, often in relation to functional impairment and psychological distress. The co-occurrence of psychiatric symptoms and chronic pain is a significant health problem, and the effect of resilience within this group has not been previously explored.

Within this study, significantly more female patients than male patients were seen at the tertiary chronic pain clinic. This is consistent with previous research demonstrating sex-linked increased chronic pain intensity (12, 13, 19). Gender bias may also be implicated, as caregivers and healthcare providers responses to expression of pain in children and youth dependent have been previously demonstrated to differ and may result in referral differences between boys and girls to tertiary care. Experimental studies have demonstrated caregivers and healthcare providers expect boys to experience more acute pain as a result of the same procedure as girls (20, 21). It is possible that girls who demonstrate prolonged intense distress or severe pain are more likely to have pain that is felt to require further assessment or support that is beyond the scope of primary care. There is evidence of sex differences in prevalence of chronic pain with co-existing depression, anxiety disorders, and Post-traumatic Stress Disorder (PTSD), with the prevalence of chronic pain comorbid with these psychiatric illnesses reportedly higher in females and increasing with age (12). This has been attributed to 5-Hydroxytryptophan signaling systems, neuroimmune interactions, and stress responses modulated by stimulatory effects of estrogen and inhibitory action of androgens on the central nervous system (22). Poorly managed acute pain and certain psychiatric symptoms appear to be predictors of formal psychiatric diagnoses of depressive and/or anxiety disorders, PTSD, and chronic pain.

Living with chronic pain has a broad impact on development, social and physical functioning, and family life (7). The effects on family life appear to be multidirectional, with family history of chronic pain appearing to be a risk factor for the development of chronic pain within this study. Furthermore, family members' psychological distress has been shown in previous studies to be linked to spreading somatic pain to new sites and result in severe chronic pain (9). Children of parents with chronic pain may have an elevated risk of developing pain-related disability in consequence of several composite interactions (23). Stone et al. (10) posited a conceptual model of intergenerational transmission of chronic pain, inclusive of genetic predisposition for heightened pain sensitivity, social learning and parental responses to pain, such as protectiveness and increased catastrophizing, as factors in chronic pain ‘transmission’ from parents to offspring (10). Dependent on type of pain, the genetic vs. environmental and social learning factors may have variable weighted impacts on the child or youth's development of chronic pain (24–26).

Social factors such as exposure to childhood trauma and socioeconomic deprivation can be risk factors as well as exacerbate the severity of chronic pain, resulting in negative effects on biopsychosocial functioning (1, 11). More than half of the children and adolescents who attended the chronic pain clinic were living below the poverty line. This is significantly more than the 9% of Canadian children under 18 years of age believed to be living below the poverty line during the study period (18). Low income, physical disability, and mental illness are often deeply intertwined (11). Such socioeconomic deprivation may further exacerbate the already complex difficulties secondary to living with chronic pain, resulting in further negative overall effects on biopsychosocial functioning (11). Therefore, a multidimensional and holistic management plan for many economically-deprived chronic pain patients should include social support interventions when clinically indicated.

Children and youth with chronic pain report more prior mental health diagnoses than the general population. Severe and/or functionally impairing chronic pain was associated with psychiatric symptoms, predominantly symptoms of excessive worry, depression and previous formal diagnoses of Generalized Anxiety Disorder and Major Depressive Disorder. Symptoms of anxiety, chronic fatigue, insomnia, and excessive worry were experienced by more than half of the study's children and youth. The relationship between psychiatric symptoms and chronic pain disorders may be conceived as bidirectional in nature. Patients with such complex problems may best be served by treatments and approaches that are systemic in nature, rather than only diagnostically-driven. This may be critical for recovery from chronic pain as other recent research suggests that mental health comorbidities and symptoms can impede recovery among youth with chronic pain (27).

Older children reported more psychiatric symptoms. This is consistent with previous longitudinal cohort studies demonstrating children with somatic complaints were more likely to develop anxiety and depressive disorders by adolescence (28).

Children with pain involving multiple pain sites endorsed greater psychiatric symptom burden. Experiencing pain located at more than one pain site has been linked with poorer emotional outcomes and increased difficulty in coping, (29). Research suggests that the coexistence of pain conditions, rather than location or type, has the largest impact on the emotional, functional, and social effects on the affected children and youth (29).

Nearly all of the patients and their families received psychoeducation related to chronic pain and the mind-body connection when they attended the clinic. The psychoeducation provided to children and youth who presented to the tertiary chronic pain clinic was intended to provide families insight into the development and maintenance of both chronic pain and psychological distress. Psychoeducation in pediatric chronic pain clinic included a message of finding ways to support patients in order to live “their best life” with pain rather than an expectation of total resolution of pain. This portends individuals and their families to have realistic expectations of recovery from severe, debilitating chronic pain. Continued participation in healthy prosocial and physical activities, rather than a reduction of activity, was strongly encouraged.

Less than half of the children and youth living with chronic pain were attending school regularly or engaging in age-appropriate prosocial activities on a regular basis. This is of major concern when considering the developmental impacts on overall health, social and physical development. This study supports integrated multidisciplinary assessment and treatment of pediatric chronic pain, which include a chronic pain specialist, psychologist, psychiatrist, physiotherapist, occupational therapist, nurse care coordinator, and social worker. Establishing an individualized and holistic management plan may reduce psychiatric symptom burden and decrease the need for pharmacological treatment, while supporting engagement in prosocial and physical activities (30). A cohesive approach to promoting wellness will support resiliency through appropriate rehabilitation in order to continue developmentally-appropriate activities.

Within the context of the current pandemic situation, children and youth with chronic pain are at greater risk of further pain-related disability and psychiatric symptoms due to inability to maintain or return to school and extracurricular activities. While complying with current public health guidelines, participating in educational and meaningful activities, maintaining a daily routine, daily exercise, and positive interaction with peers *via* virtual means should be encouraged to promote resiliency and recovery from both chronic pain and psychiatric illness.

This study has several limitations. It is cross-sectional, giving us a still life, but not capturing the longitudinal nature of living with chronic pain. It is retrospective, focusing specifically on pediatric patients who have attended the only tertiary chronic pain clinic in the province of Manitoba, Canada. On the basis of this primary methodology we confirmed the merit of future prospective longitudinal research, with systematic assessment of markers of resilience using a validated resilience scale (i.e., Connor-Davidson Resilience Scale), chronic pain, and psychiatric symptom severity in tertiary care, as well as primary care settings. This study confirmed that children and youth with chronic pain who attend a tertiary chronic pain clinic are more likely to fulfill the criteria for somatic symptom disorder as their pain symptom is very distressing and causing significant disruption in their daily lives. Future research including primary care settings would allow for better understanding of the psychiatric burden of those children and youth with chronic pain seeking support and their psychosocial function and demonstration of resilience.

Formulation of resilience in chronic pain has been limited within the literature with primary focus on illness behavior and disability. Resilience is a protective factor when it comes to chronic illness. Although it is inherent within the individual, it is enhanced through developmental, social, cultural, and environmental factors. Children and youth who exhibited resiliency through engagement in these activities demonstrated greater well-being. Specifically, they reported less psychiatric symptoms. We chose to delineate resilience from level of functioning to shift the attention to the core validation that the pain is REAL and higher level of functioning does not imply less pain. Framing school attendance and extracurricular activities as a measure of resilience integrates the common ground between treatment teams and families. Children and youth who are seeking help for chronic pain and other somatization symptoms require an acknowledgment that living with pain is excruciating. Building upon inherent individual strengths and encouraging activities that support adaptive adjustment to chronic pain within the framework of enhancing their resilience will ostensibly improve outcomes for families of children with chronic pain.

## Conclusions

This study demonstrates probable risk factors that contribute to the development of more complex chronic pain experience resulting in accessing care at a tertiary chronic pain clinic including female sex, family history of chronic pain, and socioeconomic deprivation. As the population data was drawn from reports from the provincial data repository, and the clinical data from the sole tertiary chronic pain clinic in this geographical region, there was an opportunity for direct comparative analysis of prevalence of diagnosed mental disorders and socioeconomic status of individuals who attended the clinic. Pediatric chronic pain that is severe, medically unexplained, and accompanied by psychological distress is associated with increased prevalence of previously diagnosed psychiatric disorders when compared to children within the general population. Prevalence of psychiatric symptoms of depression and anxiety, such as low mood, mood lability, irritability, amotivation, poor concentration, anergia, anhedonia, chronic fatigue, appetite disturbance, insomnia, and excessive worry and separation anxiety ranged in prevalence from 10.5 to 63.2% within the clinical sample.

There is limited previous research related to the resilience of children and youth living with chronic pain. We have endeavored to provide some insight into prosocial behaviors that are both indicative of resilience and strongly encouraged by healthcare professionals who are involved in the support and management of chronic pain patients. As children and youth who reported fewer psychiatric symptoms reported greater social function, targeting psychiatric symptoms through appropriate psychological and psychiatric management can improve outcomes.

Mental health disorders are more prevalent in patients attending chronic pain clinic than in the general population. We advocate for an integrated interdisciplinary treatment approach based on collaboration with youth and families to pay closer attention to how we can enhance resilience in youth to achieve better treatment outcomes in the future.

## Data Availability Statement

The raw data supporting the conclusions of this article will be made available by the authors, without undue reservation.

## Ethics Statement

This study involved human participants and was reviewed and approved by University Health Research Ethics Board and the Winnipeg Regional Health Authority Research Committee in Manitoba, Canada. Written informed consent from the participants' legal guardian/next of kin was not required to participate in this study in accordance with the national legislation and the institutional requirements.

## Author Contributions

MY and PA contributed to conception and design of the study. AG supported with data collection. MY performed the statistical analysis and wrote the first draft of the manuscript. AG and PA revised and wrote sections of the manuscript, and the manuscript was revised by PA. All authors contributed to manuscript revision, read, and approved the submitted version.

## Funding

This study and related open access publication fees were funded through a Department of Psychiatry Academic Project Award within the Max Rady College of Medicine, University of Manitoba, Canada.

## Conflict of Interest

The authors declare that the research was conducted in the absence of any commercial or financial relationships that could be construed as a potential conflict of interest.

## Publisher's Note

All claims expressed in this article are solely those of the authors and do not necessarily represent those of their affiliated organizations, or those of the publisher, the editors and the reviewers. Any product that may be evaluated in this article, or claim that may be made by its manufacturer, is not guaranteed or endorsed by the publisher.
